# The Holy Grail of cardiology?

**DOI:** 10.1113/EP092560

**Published:** 2025-02-13

**Authors:** Richard Godfrey

**Affiliations:** ^1^ Division of Sport, Health and Exercise Sciences Brunel University of London, London Uxbridge UK

I had been training for the last few months, getting up not long after 05.00 h to travel to my local gym to be in the pool for a 1 h swim training session at 06.00 h, four times a week. A few months earlier, some friends and I had agreed we would swim across Lake Annecy in south‐eastern France, south of Geneva. Every August the official swimming event runs, with an option of two races. La Grande Traversée is the one we were opting for; a swim of 2.4 km (Traversée du Lac d'Annecy). Training had gone well, and although there were times when I felt rough in the mornings, maybe even a little breathless, I dismissed it, and after each swim I felt fine.

Race day duly arrived. We lined up and were invited to enter the water. The gun went off and the melee began. After 10 min of bumping and scrambling, I found some space, put my head down, got into a rhythm and went for it. I finished just over 1 h later.

My friends and I were all elated, and that night we went out for a nice meal and, of course, drank too much, but the camaraderie was great, and we had a lot of fun before returning to the campsite. Fairly soon, I really wasn't feeling too good. In my one‐man tent, I couldn't sleep and felt unwell; nauseous. I crawled out of the tent and started up the modest incline towards the toilet block. My heart was racing, and the short walk and modest incline were ridiculously hard. I felt breathless. My stomach and chest were sore, as if I had bad indigestion, and I was exhausted. I didn't make the toilet block in time. I felt humiliated and embarrassed, despite the fact there was no one around to see. I cleaned myself up, returned to my tent and slept fitfully until morning.

I was feeling distinctly ‘green around the gills’, but assumed it was only a hangover, or at least had convinced myself that is all it was, but I felt uneasy because it was beyond anything I had ever experienced before. We walked along the lake shore to a restaurant for breakfast, but I couldn't face it. I ran out of the restaurant and vomited on the shore.

For the next few days I could eat nothing; in fact, even drinking water made me feel nauseous. I ate nothing and drank little or nothing for days. I was due to get the TGV back to Paris, which I did, but I didn't feel any better on the train. At Gare du Nord station, in the waiting room for the Eurostar, I felt a little breathless and was experiencing an aura. I have migraines about once every 5 years, and the visual disturbance was similar, if not exactly like the aura which had accompanied my past experiences of migraines. Sufferers often experience a visual aberration. In my case, seeing out of one eye is nothing more than visual ‘noise’; a ‘snow‐storm’ image for the whole of the right field of view, with an occasional flash of light (van Dongen and Haan, 2019).

I returned home, and for the next few weeks I seemed to recover, but training (gym, weights, cycling and swimming) did not go well. It all felt like a struggle. I wasn't getting any fitter, even when I increased the number of training sessions, and I felt tired all the time. In fact, on the bike at the gym, I was finding it difficult to drink water because it was a struggle to hold my breath even long enough to swallow.

In September, >1 month after Annecy, my alarm clock once more awoke me at 05.00 h. I felt rough. My stomach was aching, and I felt as if I had a touch of acid reflux indigestion. I hauled myself out of bed, got dressed and went to the gym. The plan was to do eight 30 s sprints on the bike. Given that during the previous few weeks, it felt as if my training was less and less effective, I had decided that improving my maximal oxygen uptake would allow me to cope with more training and to recover better from training (Tomlin & Wenger, 2001) and, of course, cope better with all the activities of daily living too. That was the reason for the bike sprints (Scribbans et al., 2016).

At the gym, I managed only five of the eight planned sprints. Feeling that my stomach was getting worse and that I was exhausted, I left the session unfinished, showered and drove the 30 min on the M40 to work. Over the next 3 h, the burning pain in my throat and stomach increased. Before long, it was bad enough that I couldn't sit still. I paced in my office and began punching myself in the face in an effort to distract from the intense burning pain in my throat and stomach (a variation on conditioned pain modulation; Enax‐Krumova et al., 2020).

Fairly soon, I couldn't stand the pain any longer, which was now in my throat, stomach and chest. I asked my colleague in the next office to take me to the Accident and Emergency department. At the hospital, merely 800 m from work, I was immediately seen, and a 12‐lead ECG was carried out (Miranda et al., 2018). Very quickly, I found myself on a gurney having a vein in each arm cannulated; one for administration of analgesics and the other for fluids. I overheard a couple of clinicians talking about ‘ST elevation’. I was shocked, and they noticed not only that had I overheard, but also that I understood what they had said. One approached, saying, ‘Just relax; you are having a heart attack, but don't worry, you are going to be fine!’.

They told me they were going to attempt to stabilize me, then transfer me to the specialist cardiac unit at Harefield Hospital, 3 miles away. Soon I was outside an ambulance for the transfer, trying to distract myself with the sights, sounds and sunshine of this warm autumn day. Very soon, however, it all went dark, and I lost consciousness. In fact, I was in cardiac arrest.

Speculation, what I was told and what I was able to deduce cover the next part of the story. I was sprinted back into the hospital and had to be defibrillated three times. The third time, the energy used was 360 J, sufficient to leave burn marks around the edges of the gel pads between the defibrillation paddles and my skin. I had been in ventricular fibrillation for 6 min before that final shock worked (Adgey, Spence and Walsh, 2005).

I had no near‐death experience, but as I travelled the long and arduous journey back to sentience I was aware of (and would publish later on) a number of stages (Figure 1). I wrote an autoethnography on the whole heart attack experience, which is unpublished but which describes that journey as trying to ‘swim through treacle with an anchor tied to your ankles’.

**FIGURE 1:**
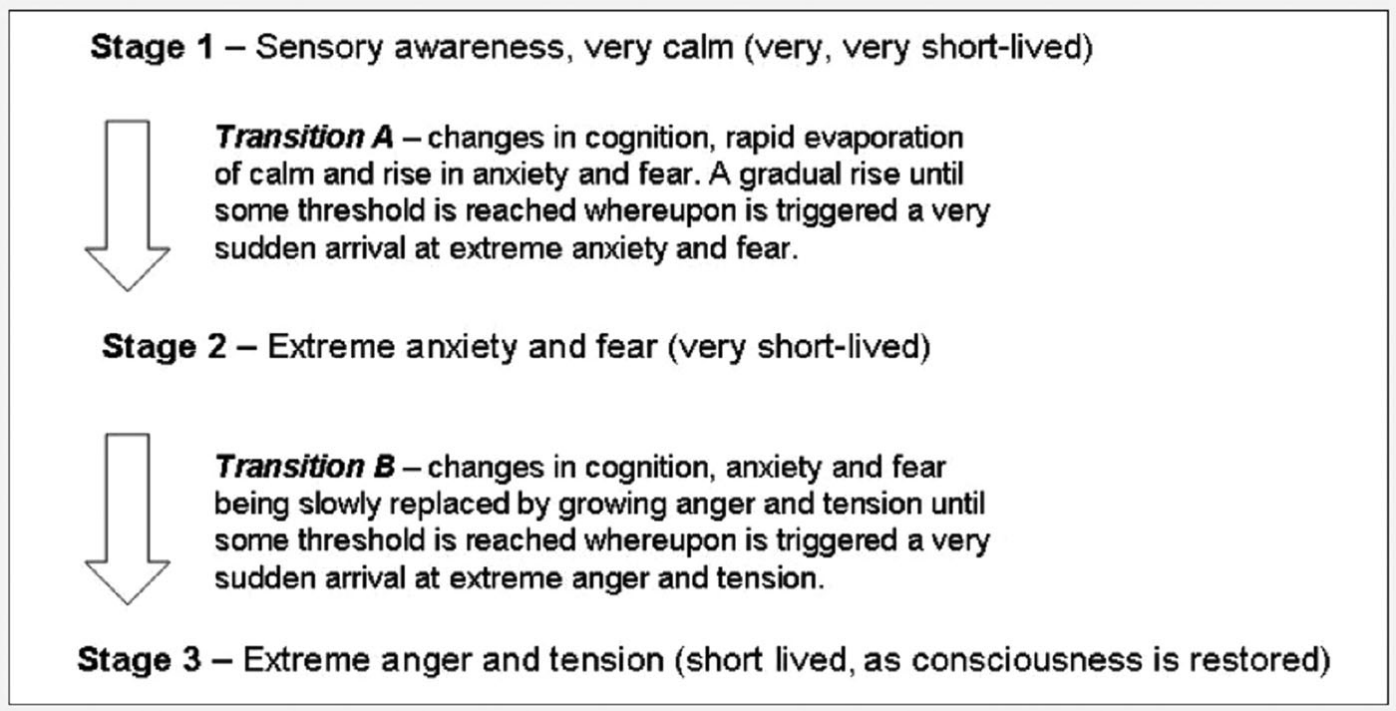
Three stages after defibrillation between total lack of awareness and full sentience/recovery of consciousness (Adapted from Lane and Godfrey, 2010).

These stages are described in Figure 1, and the extreme anger experienced in Stage 3 was shocking by the extent to which I was completely controlled by it. I suggest this might be an extreme surfacing of Darwinian survival mechanisms (Lane and Godfrey, 2010), but it might best fit the Carver and Harmon‐Jones psychological model for an individual ‘in limbo’, between unconsciousness and full consciousness (Carver & Harmon‐Jones, 1997).

In the ‘Cath Lab’ (basically, an operating theatre) at Harefield Hospital (Figure 2), a large blood clot was discovered in the right coronary artery, which was aspirated. Subsequently, the vessel was confirmed as still patent 48 h later (Figure 3).

**FIGURE 2:**
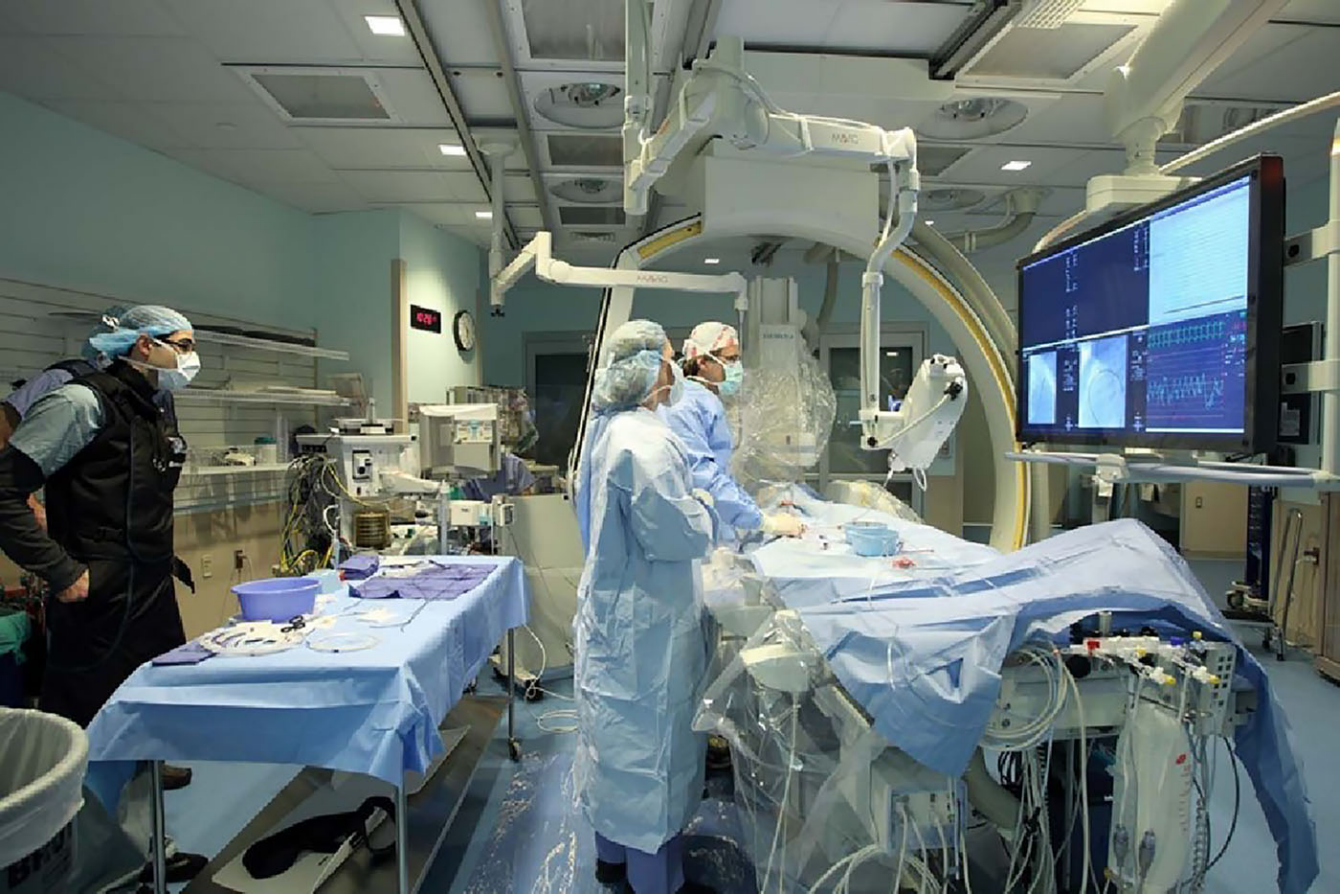
A hospital ‘Cath Lab’. At least two clinicians insert a catheter via the femoral artery and feed the catheter up into the right coronary artery, where contrast medium (dye) is injected to highlight the line of the vessel and any obstruction.

**FIGURE 3:**
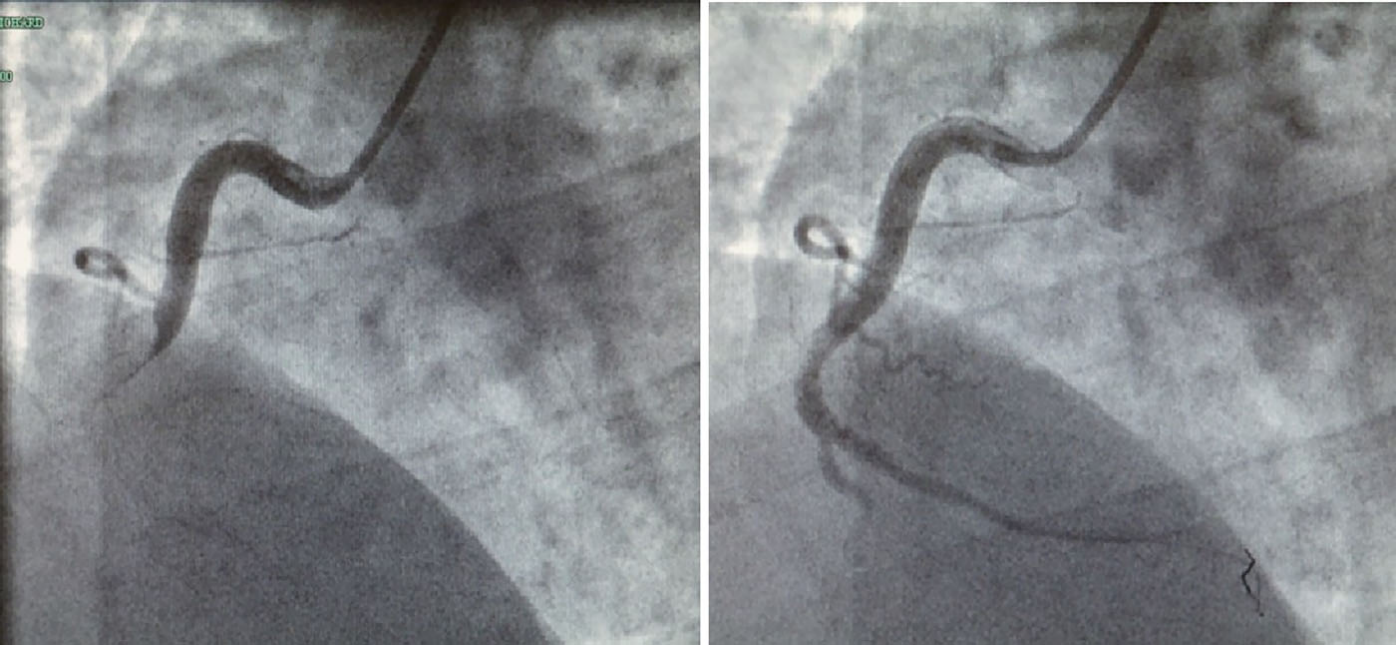
Left‐hand image shows a blockage in the right coronary artery resulting in ischaemia in a downstream locus of the left ventricle. Right‐hand image shows that the right coronary artery is still patent 48 h following aspiration.

The consequence of vessel occlusion is ischaemia (inadequate blood flow) in the myocardial tissue downstream of the occlusion and subsequent necrosis (death and infiltration of scar tissue; Figure 4).

**FIGURE 4:**
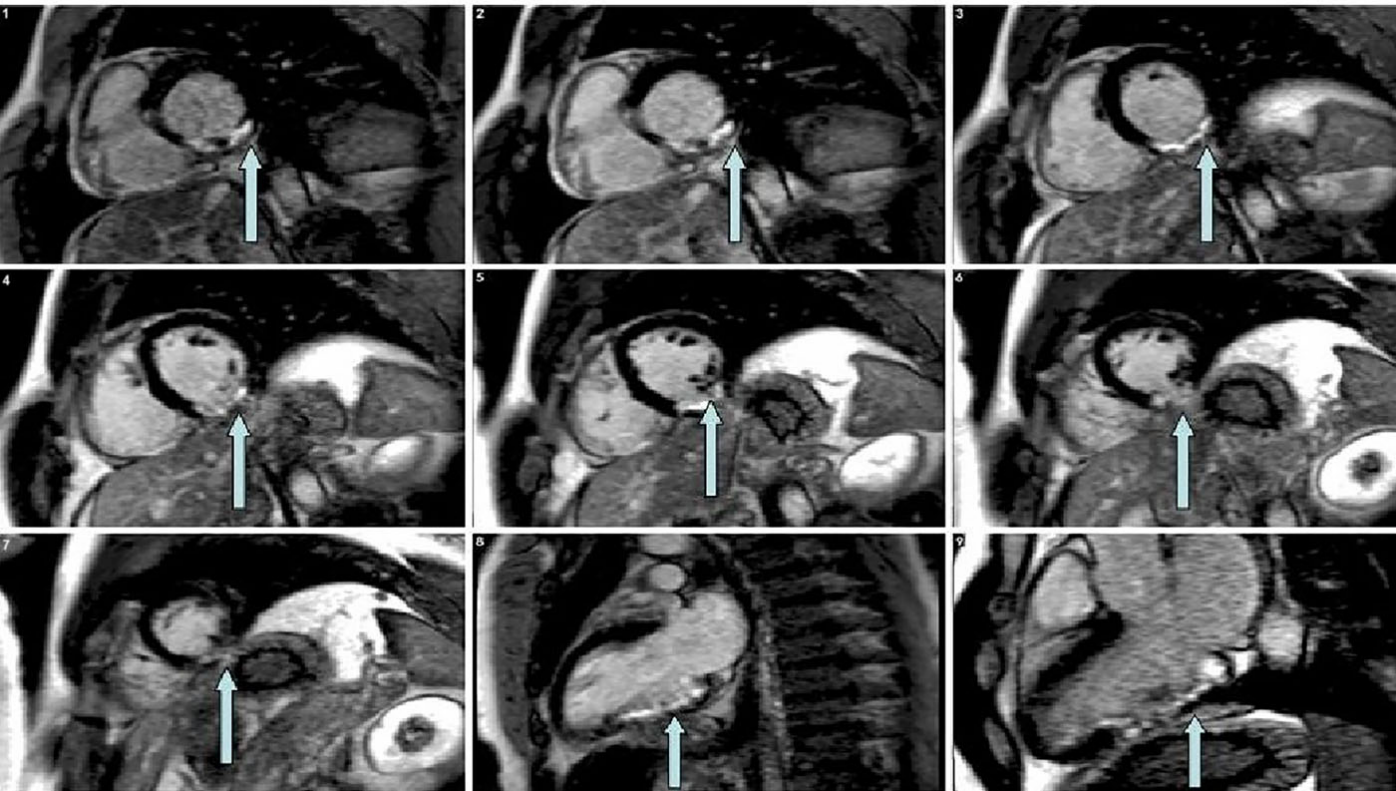
Cardiac functional MRI, with multiple images from different angles and with arrows pointing to scarring amounting to 16.3% of the left ventricle.

The myocardial infarction (MI) occurred with no real prior indication and although I had no family history of heart disease or MI in first‐degree relatives (parents/sibling), few modifiable risk factors (non‐smoker, moderate drinker, reasonably good diet and regular exerciser) would suggest low risk for MI. I was only 45 years old at the time, and in terms of exercise, I did quite a lot. I kept a training diary and, by that time, had regularly exercised hard for >30 years; that is, long‐term exercise, which included a mixture of 20 years of karate, regular squash, swimming, cycling, running and weight training, which amounted to 416 weeks without a break (two to four sessions of resistance exercise per week for 8 years). The day before the MI, I did a 3600 m swim session in slightly >60 min, and within 14 days afterwards, I was back in the pool and did 10 × 200 m (Whyte et al., 2009). This sounds radical, but it is important to exercise ideally within a week of an MI to ensure that remodelling (chaotic organization of cardiac myofibrils) can be minimized (Haykowsky et al., 2013)

Unfortunately, it was not clear why I had an MI. A definitive diagnosis was not possible, despite follow‐up angiographies 2 days and 6 months later. Almost 2 years later, at home on my own and having had an aching, warm and slightly swollen right leg all night, I knew something was not right. As I was coming down the stairs in the morning, I passed out and regained consciousness, I have no idea how much longer afterwards, at the foot of the stairs on the hall carpet. I felt very hot and clammy and very breathless as I called for an ambulance whilst wiping blood from my lip, which was cut by a tooth when I hit the carpet.

After a whole day of hospital tests, including blood gases and so forth, a CT scan identified many clots in both my right leg and throughout the lung vasculature. Well, that would explain the breathlessness. The risks of giving me a thrombolytic agent (alteplase, a plasminogen activator; Jilani and Siddiqui, 2024) were considered carefully. The conflicting risks are that of haemorrhage with alteplase versus failure of passive resolution of bilateral pulmonary emboli with time. As a young, otherwise healthy individual, it was decided I should have the intravenous ‘clot‐busting’ medication, administered in accordance with the British Thoracic Society Guidelines (BTS Guidelines, 2005). Within an hour of receiving alteplase, my day of uncomfortable breathlessness was gone. Figure 5 shows blood clots in lung vasculature (white dots), and the ‘back pressure’ in the pulmonary artery has caused the heart to be ‘inflated’ and ‘rounder’ than it would be in normal physiological conditions.

**FIGURE 5:**
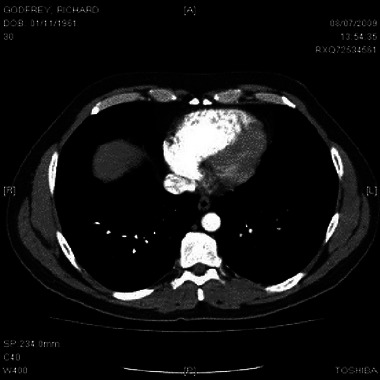
CT pulmonary angiography (transaxial view), demonstrating bilateral pulmonary emboli (blood clots, seen here as white dots following the lines of the pulmonary arteries) in the vasculature of both lungs and a misshapen heart attributable to raised pulmonary artery pressure causing ‘back pressure’. The grey ‘smudge’ to the right of the heart (i.e. to the left as shown in the image, because this transaxial slice is viewed from the inferior position by convention) is the top of the right hemidiaphragm. At the top of the image are the pectoral muscles. The large white body near the bottom of the image is a thoracic vertebra.

Far from exhaustive testing meant that there was still no definitive diagnosis. As a physiologist, I read as much as I could about my symptoms over the next 6 months. No one else seemed to have spotted the fact that I seemed to have many of the signs and symptoms of Hughes syndrome [named after its discoverer Graham Hughes, in 1983 and also known as antiphospholipid syndrome (APLS); Hughes, 2008].

By March, 8 months after my pulmonary emboli and 2.5 years after my MI, I still did not have a diagnosis from a qualified medic. From my reading of the literature, APLS seemed the most plausible diagnosis, but referral to the haematologist at the local hospital was very unsatisfactory. He seemed too intent on showing off to a female medical student to take an adequate history. In addition, I had come to the hospital straight from doing weight training at the gym, and he insisted on asking me three times if I took anabolic steroids. After this experience, I self‐referred to the private London Bridge Clinic where Graham Hughes’ team worked. I tested negative for the three antibodies known to be markers for APLS. Despite that, the private consultant diagnosed me with APLS because all the other signs and symptoms were strongly indicative of that condition. One of these strong indicators was a venous thromboembolus (blood clots) in the right coronary artery at 45 years old or younger; basically, a ‘tick in both boxes’. In the absence of any of the three antibodies, the consultant suggested that, as a relatively new condition, I could easily have another antibody not yet identified as a marker of APLS. Regardless, I fulfilled the rest of the criteria of the Sapporo Classification for APLS/Hughes syndrome (Miyakis et al., 2006), and my treatment would be daily oral warfarin for life (Godfrey et al., 2011).

Now I had a definitive diagnosis, but cardiac consultants were telling me ‘no heroics’ regarding my subsequent exercise and that once acquired, myocardial scar is ‘permanent and immutable’. This is often stated and justified on the basis that the adult human heart is a postmitotic organ and cannot be repaired. Even at that time, this was considered to be a controversial statement (Anversa et al., 2006,). As an exercise physiologist, I found this somewhat unlikely and not particularly believable. Once more, I did a few more months of reading on the effect of exercise on cardiac function. There was a lot of research on high‐intensity interval training and the benefits to heart function, even in the face of certain pathologies, with no suggestion that high‐intensity interval training was any more risky than lower exercise intensities (Ito, 2019). At the time, there was little on high‐intensity interval training and repair of human hearts damaged by MI, which was another reason that encouraged me to try this mode.

Some of the literature speculated on the emerging findings in stem cells, which included some very interesting findings on the effects of vigorous exercise in activating stem cells of rats and mice with damaged myocardia and the consequent repair achieved (Waring et al., 2014). I came across the review by Wisloff et al. (2009), which suggested that vigorous exercise in the form of 4 × 4 min at >90% of maximal heart rate (with 3 min rest intervals) was demonstrated to be effective for human heart function. At the time, there were no findings to demonstrate that cardiac repair could be affected by high‐intensity/vigorous exercise in humans post‐MI. Improved function? Yes, but all evidence at that time was on animal models, not on humans, and there was nothing on cardiac repair in humans.

In general, there is very little research on cardiac regeneration and repair, but why might this be an important area to examine? Well, those who have had an MI incur heart damage as a consequence, even if there was no extant disease of the heart or coronary vessels before the MI. There is myocardial damage and scar tissue infiltration as a consequence of the MI.

The extent of scar tissue after a first MI is related to the risk of subsequent events; more scar, more chance of arrhythmia, hence a greater chance of another MI (Li, 2019). Prior MI also increases the risk of venous thromboembolus by 20% (Smeeth, 2006). Of course, I wanted to reduce my own risk of having another near‐fatal or fatal episode, but I also considered the cost of care for MI survivors to the National Health Service, the consequences for victims and their families, and the potential to make a real contribution to science and medicine. The effect on the survivor's family can be significant. Weeks after my MI, I visited my dad in Glasgow. When he opened the door I went to hug him, but he couldn't and instead burst into tears. For survivors too, there can be lasting psychological effects ranging from slight cognitive dysfunction to post‐traumatic stress disorder, something that has been known for >30 years (Doerfler et al., 1994).

For many years I also used my own MI in my own University teaching, presenting it as an anonymous case study, including the patient's long history of engagement with exercise. I then asked the audience of students: do you think this demonstrates that exercise is a waste of time or that without exercise this person would have died? All hands go up for the latter, and I then reveal that I am the patient in question. It always caused a real stir and I still, occasionally, have former students stop me in the street, years after they have graduated, to tell me how impactful it was and that it remains one of the most memorable things from their time at university.

As a long‐term exerciser, familiar with pushing myself hard, I already knew that the 4 × 4 min protocol was almost impossible. Most normal people cannot cope with 4 min efforts at an intensity designed to elicit >90% maximal heart rate for a sustained part of the interval; it is far too hard. However, some research has also examined the use of 1 min intervals (Currie et al., 2013). This seemed much more manageable to me. I opted for 1 min efforts, and over a few weeks built up to doing 10 × 1 min efforts of sufficient intensity to elicit a heart rate of >90% maximum for ≥25% of the ‘work interval’, alternating with 1 min active recovery at an easy workload. I did this three times per week for 60 weeks. The training data for each month are shown in Table 1.

**TABLE 1:** Number and volume of sessions of high‐intensity interval training per month.

Training	Jan	Feb	Mar	Apr	May	Jun	Jul	Aug	Sep	Oct	Nov	Dec	Jan	Feb
AHIT (*n*)	12	8	15	13	6	8	14	9	6	10	6	7	6	9
AHIT (min)	410	285	410	474	220	254	563	280	185	285	245	272	240	366

*Note*: Please note the term ‘AHIT’ is used in Table 1 which is exactly equivalent to ‘HIIT’ which is used throughout the rest of this current editorial.

Functional cardiac MRI data were collected in November 2008, May 2011 and March 2012 (referred to in Figure 6 as Study 1, 2 and 3, respectively) with a full set of cine to assess delayed postcontrast images for scar evaluation. This is not entirely obvious from Figure 6 as these are still images from the original functional (cine / moving) images but are included for full disclosure as Figure 6 (provided from Godfrey et al., 2013).

**FIGURE 6:**
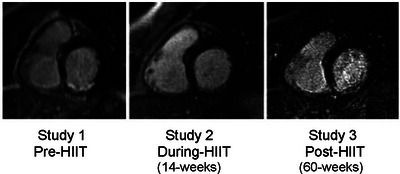
Three still images, each taken from functional cardiac MRI (i.e. cine images). Data collected were Study 1, November 2008; Study 2, May 2011; and Study 3, March 2012.

I suggest that the intervention of high‐intensity exercise three times per week for 60 weeks played a role in reducing my scar tissue by 48%, from 16.3 to 7.8% of the left ventricle (Godfrey et al., 2013). Of course, a role for spontaneous recovery cannot be discounted. In all probability, exercise and spontaneous recovery two combined to result in the cardiac repair that is presented here.

In 2011, the British Heart Foundation launched its ‘Mending Broken Hearts Appeal’, and there was much talk of finding the ‘Holy Grail of cardiology’; that is, a treatment to ‘mend broken hearts’, including research on stem cells and the drugs that might activate them. I have little doubt that since 2011 there has been sustained research, with attendant huge funding, for potential drug treatments. Research on exercise lags far behind. Perhaps there is now a need, especially in the light of recent problems in the National Health Service, for an improved balance, in which funding to address the role of exercise, in both prevention and treatment, is examined more seriously and in greater balance with the focus on drug treatment. Who knows, but perhaps it is exercise that will prove to be the Holy Grail of cardiology!

## AUTHOR CONTRIBUTIONS

Sole author.

## CONFLICT OF INTEREST

None declared.

## FUNDING INFORMATION

None.
